# Differential associations of cardio-metabolic diseases by population group, gender and adiposity in South Africa

**DOI:** 10.1371/journal.pone.0202899

**Published:** 2018-09-27

**Authors:** Nasheeta Peer, Yusentha Balakrishna, Anniza de Villiers, Pamela Naidoo

**Affiliations:** 1 Non-communicable Diseases Research Unit, South African Medical Research Council, Durban, South Africa; 2 Department of Medicine, University of Cape Town, Cape Town, South Africa; 3 Biostatistics Unit, South African Medical Research Council, Durban, South Africa; 4 Non-communicable Diseases Research Unit, South African Medical Research Council, Cape Town, South Africa; 5 Heart and Stroke Foundation South Africa, Cape Town, South Africa; 6 Stellenbosch University, Cape Town, South Africa; University of Perugia, ITALY

## Abstract

**Aims:**

To describe the distribution and examine the associations of diabetes, hypertension and hypercholesterolaemia across and within population groups, gender and body mass index (BMI) categories.

**Methods:**

This national cross-sectional study was conducted in 2013 among ≥18-year-old black African, coloured, white and Indian adults self-selected for screening. Data collection included self-reported behavioural risk factors and clinical measurements comprising blood pressure, anthropometry and point-of-care random blood glucose and cholesterol assessments.

**Results:**

Among the 7711 participants, 2488 men and 5223 women, the prevalence of diabetes and hypertension increased by BMI category across population groups. Compared with white men and women, black African men (odds ratio: 2.66, 95% confidence interval: 1.70–4.16) and women (2.10, 1.49–2.96), coloured men (2.28, 1.44–3.60) and women (2.15, 1.52–3.05) and Indian men (4.38, 2.65–7.26) and women (3.64, 2.50–5.32) were significantly more likely to have diabetes. The odds for hypertension were significantly higher only in coloured men compared with white men (1.37, 1.02–1.83), while it was significantly higher in black African, coloured and Indian women compared with white women. The odds for hypercholesterolaemia were significantly lower in black African men (0.64, 0.49–0.84) and women (0.52, 0.43–0.62) compared with white men and women, and significantly higher in Indian men (1.47, 1.05–2.08) compared with white men. Black African women compared with their male counterparts were less likely to have diabetes (0.64, 0.46–0.89). Black African (0.66, 0.54-.082), coloured (0.65, 0.50–0.84) and white (0.69, 0.53–0.88) women were significantly less likely to have hypertension compared with their male counterparts. The odds for hypercholesterolaemia were higher in coloured (1.44, 1.16–1.80) and white (1.47, 1.18–1.84) women compared with their counterparts.

**Conclusions:**

The cardio-metabolic diseases of diabetes, hypertension and hypercholesterolaemia were differentially associated with population groups and gender in South Africa. The insights obtained highlight the need for multi-disciplinary targeted management approaches in high-risk populations.

## Introduction

Globally, changing behavioural patterns with increasing intakes of caloric-rich diets, decreases in physical activity levels, and alcohol and tobacco use are key contributors to the development of cardiovascular diseases (CVDs), including type 2 diabetes mellitus (hereafter referred to as diabetes), hypertension, hypercholesterolaemia and obesity. However, studies have shown that environmental, nutritional, lifestyle and genetic influences, possibly together with other known or unknown factors, may differentially influence the development of CVDs in certain ethnic or population groups [1].

Numerous studies from high-income countries have examined and reported differences in the risk for CVDs across population groups or ethnicities [2–6]. The populations or ethnicities reported on have include African-American, Hispanic/Mexican, Asian, and Aborigine and Maori communities, among others. Mainly, these studies have examined non-white populations who may have been ethnic minorities in terms of race, language, nationality, culture and/or religion, or foreign-born.

South Africa, as a result of the legacy of apartheid, and unique to the country, has four previously defined official population groups, and no recent studies examining differences in the risk for CVDs across and within these population groups. Some comparative studies were conducted over 20–30 years ago usually in small samples that did not include all four population groups i.e. black African, coloured or mixed ancestry, white and Indian [7, 8]. Nevertheless, the prevalence of diabetes, hypertension, hypercholesterolaemia and obesity in South Africa are high and increasing [9], and warrant attention. Obesity, in particular, which is closely linked to the development of the other cardio-metabolic diseases, has reached epidemic proportions in women.

Identifying population groups at high risk for specific cardio-metabolic diseases may lead to the development of targeted CVD screening, prevention and management programmes. These may be more cost-effective in reducing diabetes and hypertension complications and CVD events such as heart attacks and strokes. Therefore, this study aims to describe the distribution and examine the associations of diabetes, hypertension and hypercholesterolaemia across and within population groups, gender and body mass index (BMI) categories.

## Methodology

### Study design and sampling procedure

The Heart and Stroke Foundation South Africa (HSFSA) conducted this cross-sectional study in 2013 in five of the nine South African provinces among ≥18-year-old adults self-selected for screening. These comprised eight sites each in Western Cape and KwaZulu-Natal, seven sites in Gauteng, five in Eastern Cape and one in Free State. Logistical considerations in terms of transportation and accommodation costs dictated the selection of sites and provinces for screening. These were situated mainly within urban and semi-urban settings; rural settings were not included due to a lack of resources and the large geographical distances that would have needed to be covered. Venues with greatest exposure to the public such as shopping malls, church halls, community centres, wellness centres and schools were selected for the screenings. Community newspapers and radio stations, and the HSFSA website (www.heartfoundation.co.za) were used to raise awareness of the screening sessions. About 85 participants were screened per day with the screenings conducted in local languages including Xhosa, Zulu, English and Afrikaans.

### Data collection

Nursing professionals who conducted the fieldwork received about two weeks of intensive training, including post-training assessments of their knowledge. The team consisted of 12 nurses with three conducting each screening session.

Adult volunteers who presented at the testing sites provided written informed consent to be screened and completed the HSFSA’s “Cardiovascular Health Check” form. Self-reported data collected included demographic profiles, relevant personal and family medical history, intake of fruit and vegetables, and foods high in salt and fats, and history of physical activity levels, smoking and alcohol use.

Clinical assessments included blood pressure (BP) and anthropometric measurements. Using an appropriately sized cuff and with the participant seated and relaxed, BP was taken twice using an Omron M2 digital monitor. Participants wore light clothing and stood barefoot to determine their weight to the nearest 0.1 kg and their height to the nearest 0.1 cm. A regularly calibrated portable scale and a stadiometer were used to obtain the respective measurements.

Point-of-care biochemical assessments for random blood glucose (RBG) and total cholesterol levels were conducted using an Accutrend glucometer and an Accuchek Active machine, respectively.

### Definitions

Participants were categorised according to the previously defined official South African population groups. In 2013, this comprised 79.8% black African, 9.0% coloured, 8.7% white and 2.5% Indian [10].

Cardiac illness, either personal or family medical history, was defined as having a heart attack, angina, cardiac surgery or any other cardiac problems. An early onset cardiac problems or stroke described a paternal or maternal history of cardiac illness or stroke before the age of 55 years or 65 years, respectively.

Hypertension, diabetes, hypercholesterolaemia and adiposity were defined using standardised, international cut-off values [11–14]. Categories of adiposity were determined using BMI, calculated as the individual’s weight in kilograms divided by their height in metres squared (kg/m^2^). Overweight was defined as 25–29.9 kg/m^2^, obesity as ≥30 kg/m^2^ and underweight as <18.5 kg/m^2^ [14]. Using the average of the two BP readings, hypertension was defined as systolic BP ≥140 mmHg and/or diastolic BP ≥90 mmHg or the use of antihypertensive agents [11]. A history of known diabetes or RBG ≥11.1 mmol/l were considered suggestive of diabetes [12] while RBG 7.0–11.0 mmol/l was classified as impaired glycaemia. A total cholesterol ≥5 mmol/l defined hypercholesterolaemia [13]. Diabetes and hypercholesterolaemia medication history were not collected and, therefore, are not included in the definition of these conditions.

### Data analysis and statistical interpretation

Data were analysed using Stata version 14 (StataCorp., College Station, TX, USA). Associations between population groups (Tables 1 and 2), BMI categories (S1 and S2 Tables) and demographic factors, lifestyle behaviours, food intake and self-reported medical history in men and women were explored using the chi-square test or Fisher’s exact test where applicable. Differences in mean BP, RBG and total cholesterol levels across population groups and BMI categories were explored using ANOVA.

**Table 1:** Socio-demographic characteristics (N, %), lifestyle behaviours and medical history presented by population group and gender.

	Men					Women				
Population group	Black African	Coloured	White	Indian	p-value	Black African	Coloured	White	Indian	p-value
Number, %	**883 (35.5)**	**503 (20.2)**	**529 (21.3)**	**432 (17.4)**		**1884 (36.1)**	**1270 (24.3)**	**1070 (20.5)**	**806 (15.4)**	
**Socio-demographic**										
Mean age, SD	40.4 (13.3)	47.3 (15.1)	54.4 (17.8)	52.1 (15.9)	<0.001	42.2 (14.8)	48.5 (14.9)	55.1 (18.1)	53.7 (14.9)	<0.001
**Province**					<0.001					<0.001
Western Cape	262 (29.7)	364 (72.4)	216 (40.8)	29 (6.7)		688 (36.5)	1048 (82.5)	380 (35.5)	47 (5.8)	
Eastern Cape	111 (12.6)	79 (15.7)	155 (29.3)	8 (1.9)		201 (10.7)	120 (9.5)	320 (29.9)	7 (0.9)	
Kwazulu-Natal	120 (13.6)	13 (2.6)	61 (11.5)	338 (78.2)		317 (16.8)	45 (3.5)	153 (14.3)	695 (86.2)	
Free State	84 (9.5)	19 (3.8)	11 (2.1)	1 (0.2)		112 (5.9)	11 (0.9)	32 (3.0)	0 (0.0)	
Gauteng	306 (34.7)	28 (5.6)	86 (16.3)	56 (13.0)		565 (30.0)	46 (3.6)	185 (17.3)	57 (7.1)	
**Lifestyle behaviours**										
Physical activity >150 min/week	405 (45.9)	208 (41.4)	309 (58.4)	249 (57.6)	<0.001	816 (43.3)	513 (40.4)	538 (50.3)	425 (52.7)	<0.001
Smoking	211 (23.9)	150 (29.8)	88 (16.6)	130 (30.1)	<0.001	121 (6.4)	297 (23.4)	172 (16.1)	72 (8.9)	<0.001
Alcohol use	430 (48.7)	164 (32.6)	267 (50.5)	119 (27.6)	<0.001	404 (21.4)	228 (18.0)	420 (39.3)	60 (7.4)	<0.001
Problem drinkers^a^	427 (99.3)	163 (99.4)	267(100.0)	119(100.0)	<0.001	401 (99.3)	223 (97.8)	419 (99.8)	60 (100.0)	<0.001
**Food intake**										
≥5 fruit & vegetables/day	476 (53.9)	227 (45.1)	252 (47.6)	237 (54.9)	0.002	1206 (64.0)	609 (48.0)	589 (55.1)	512 (63.5)	<0.001
High fat foods	563 (63.8)	315 (62.6)	205 (38.8)	232 (53.7)	<0.001	1191 (63.2)	642 (50.6)	266 (24.9)	348 (43.2)	<0.001
High salt foods	595 (67.4)	295 (58.7)	190 (35.9)	199 (46.1)	<0.001	1259 (66.8)	663 (52.2)	278 (26.0)	248 (30.8)	<0.001
**Personal medical history**										
Cardiac/stroke	52 (5.9)	64 (12.8)	97 (18.3)	85 (19.7)	<0.001	146 (7.8)	174 (13.7)	169 (15.8)	142 (17.6)	<0.001
Diabetes	71 (8.0)	51 (10.1)	45 (8.5)	101 (23.4)	<0.001	167 (8.9)	142 (11.2)	72 (6.7)	203 (25.2)	<0.001
Hypertension	155 (17.6)	144 (28.6)	171 (32.3)	151 (35.0)	<0.001	444 (23.6)	412 (32.4)	331 (30.9)	352 (43.7)	<0.001
**Family medical history**^b^										
Father	41 (4.6)	58 (11.5)	55 (10.4)	51 (11.8)	<0.001	110 (5.8)	156 (12.3)	146 (13.6)	94 (11.7)	<0.001
Mother	49 (5.6)	54 (10.7)	30 (5.7)	36 (8.3)	0.001	133 (7.1)	162 (12.8)	109 (10.2)	104 (12.9)	<0.001

^a^Among alcohol consumers, men who drank ≥2 units/day or women who drank ≥1 unit/day

^b^Any cardiac problem or stroke in a father before 55 years of age or in a mother before 65 years of age

**Table 2:** Cardio-metabolic risk factors presented by population group and gender*.

	Men					Women				
Population group	Black African	Coloured	White	Indian	p-value	Black African	Coloured	White	Indian	p-value
Number	**883 (35.5)**	**503 (20.2)**	**529 (21.3)**	**432 (17.4)**		**1884 (36.1)**	**1270 (24.3)**	**1070 (20.5)**	**806 (15.4)**	
**Body mass index (BMI)**					<0.001					<0.001
Underweight, %	21 (2.4)	11 (2.2)	6 (1.1)	17 (3.9)		14 (0.7)	16 (1.3)	28 (2.6)	20 (2.5)	
Normal weight, %	312 (35.5)	128 (25.6)	134 (25.4)	174 (40.3)		329 (17.5)	267 (21.0)	411 (38.6)	256 (31.7)	
Overweight, %	326 (37.0)	199 (39.7)	237 (44.9)	165 (38.2)		447 (23.8)	388 (30.5)	337 (31.7)	289 (25.8)	
Obese, %	221 (25.1)	163 (32.5)	151 (28.6)	76 (17.6)		1091 (58.0)	599 (47.2)	288 (27.1)	241 (29.9)	
Mean BMI, SD (kg/m^2^)	27.1 (5.4)	28.2 (5.3)	28.1 (4.9)	26.2 (5.3)	<0.001	32.1 (7.3)	30.5 (6.6)	27.3 (6.1)	27.6 (5.6)	<0.001
**Blood pressure (BP)**										
Mean systolic BP, SD (mmHg)	134.5(17.2)	138.2(19.3)	136.3(16.4)	135.2(18.3)	0.002	130.7(19.8)	133.8(20.5)	131.6(18.6)	136.6(22.4)	<0.001
Mean diastolic BP, SD (mmHg)	78.7(11.8)	79.2(11.9)	77.9(11.3)	81.2(12.3)	<0.001	78.7(12.0)	77.5(11.3)	75.5(10.3)	79.6(12.1)	<0.001
SBP ≥140 mmHg or DBP ≥90 mmHg or on treatment, %	384 (43.5)	272 (54.1)	298 (56.3)	248 (57.4)	<0.001	794 (42.1)	642 (50.6)	506 (47.3)	498 (61.9)	<0.001
Known hypertension on treatment,%**	155 (40.4)	144 (52.9)	171 (57.4)	151 (60.9)	<0.001	444 (55.9)	412 (64.2)	331 (65.4)	352 (70.7)	<0.001
% of known hypertension with BP <140/90 mmHg	68 (43.9)	60 (41.7)	73 (42.7)	62 (41.1)	0.963	197 (44.4)	200 (48.5)	161 (48.6)	139 (39.5)	0.040
**Random blood glucose (RBG)**										
Mean RBG, SD (mmol/l)	6.2 (2.8)	6.4 (2.4)	6.2 (2.0)	7.3 (3.3)	<0.001	6.2 (2.6)	6.4 (2.5)	6.0 (1.5)	7.1 (3.6)	<0.001
RBG: 7.0–11.0 mmol/l	143 (16.2)	87 (17.3)	98 (18.5)	106 (24.5)	0.003	257 (13.6)	229 (18.0)	144 (13.5)	166 (20.6)	<0.001
RBG ≥11.1 or known diabetes, %	85 (9.6)	63 (12.6)	50 (9.5)	113 (26.2)	<0.001	182 (9.7)	155 (12.2)	73 (6.8)	216 (26.8)	<0.001
Newly diagnosed diabetes, %	14 (16.5)	12 (19.0)	5 (10.0)	12 (10.6)	<0.001	15 (8.2)	13 (8.4)	1 (0.7)	13 (7.8)	<0.001
Known diabetes, %**	71 (83.5)	51 (81.0)	45 (90.0)	101 (89.4)	<0.001	167 (91.8)	142 (91.6)	72 (98.6)	203 (94.0)	<0.001
% of known diabetes with RBG <7.0 mmol/l	34 (47.9)	16 (31.4)	16 (35.6)	24 (23.8)	0.011	58 (34.7)	52 (36.7)	36 (50.0)	64 (31.5)	0.045
**Total cholesterol (TC)**										
Mean TC, SD (mmol/l)	4.4 (1.0)	4.8 (1.2)	4.9 (1.3)	4.7 (1.4)	<0.001	4.5 (1.1)	5.1 (1.3)	5.1 (1.3)	4.7 (1.3)	<0.001
TC >5 mmol/l, %	195 (22.1)	187 (37.3)	216 (40.8)	139 (32.2)	<0.001	476 (25.3)	605 (47.7)	521 (48.7)	257 (31.9)	<0.001
**Prevalence of any of the 3 cardio-metabolic abnormalities**					<0.001					<0.001
1 abnormality	333 (37.8)	221 (44.0)	232 (43.9)	185 (42.8)		674 (35.9)	486 (38.3)	489 (45.7)	309 (38.4)	
2 abnormalities	135 (15.3)	126 (25.1)	151 (28.6)	120 (27.8)		298 (15.9)	375 (29.6)	268 (25.1)	239 (29.7)	
3 abnormalities	20 (2.3)	16 (3.2)	10 (1.9)	25 (5.8)		60 (3.2)	55 (4.3)	25 (2.3)	61 (7.6)	

*Data presented as N (%) or mean (SD)

**Among those with hypertension or diabetes, the proportion that was known

A univariable logistic regression was performed for each variable (age, province, family and personal medical history, diet relating to fruit and vegetable, salt and fat intake, physical activity, smoking, alcohol misuse, adiposity, and gender and population group where relevant) to determine the effect on the odds of hypertension, diabetes and hypercholesterolaemia by population groups and gender. Variables with a p-value of ≤0.05 from the univariable analyses were then used to construct the multivariate models for each population group and gender. The results for gender, population group and BMI category are presented as odds ratios (ORs) with the corresponding 95% confidence intervals (CIs). Results were considered significant for p <0.05.

For the variable population group, “white” was used as the reference to allow for comparability with international studies. There was an additional category for population group titled “other”, which was not included in the analyses because it comprised a small number of participants. However, the exclusion of this category did not affect the study findings.

All participants provided written informed consent. The South African Medical and Research Council’s Research and Ethics Committee approved the study.

## Results

### Distribution of CVD risk factors in men and women

Among the 7711 participants, 2488 men and 5223 women, mean ages were 47.6 years and 48.6 years, respectively, with White and Indian men and women significantly older than their black African and coloured counterparts **(Table 1)**. There were significant differences in lifestyle risk factors across population groups in both men and women. Smoking was most prevalent in Indian men (30.1%) and coloured women (23.4%). Alcohol use was most common in white (50.5%) and black African (48.7%) men, and in white women (39.3%). Black African men and women were most likely to consume high fat and salt diets compared with their counterparts.

Obesity levels were higher in women than in men (43.7% vs. 25.7%, p<0.001) **(S1 Table)**. By population group, obesity was most prevalent in coloured men (32.5%), and in black African (58%) and coloured (47.2%) women compared with their counterparts **(Table 2)**.

Unsurprisingly, the prevalence of hypertension, diabetes and hypercholesterolaemia increased across BMI categories in both men and women peaking in the obese, except for hypercholesterolaemia in women, which was highest in overweight women **(S2 Table)**. That the lowest hypertension prevalence by population group was in black African men (43.5%) and women (42.1%) may partly be due to their younger mean ages **(Table 2)**. Notably, black African men (40.4%) and women (55.9%) with hypertension were least likely to be on hypertension medication compared with their counterparts (p <0.001 for both). However, although Indian men and women with hypertension were most likely to be treated, they were least likely to be controlled on treatment (41.1% and 39.5%, respectively) but this was not significantly different in men. Management of diabetes was also poor among those with known diabetes. Indian men and women with known diabetes had significantly lower levels of RBG <7.0 mmol/l (23.8% and 31.5%, respectively) than their counterparts.

### Distribution of cardio-metabolic diseases by BMI category and population groups

**Fig 1:**
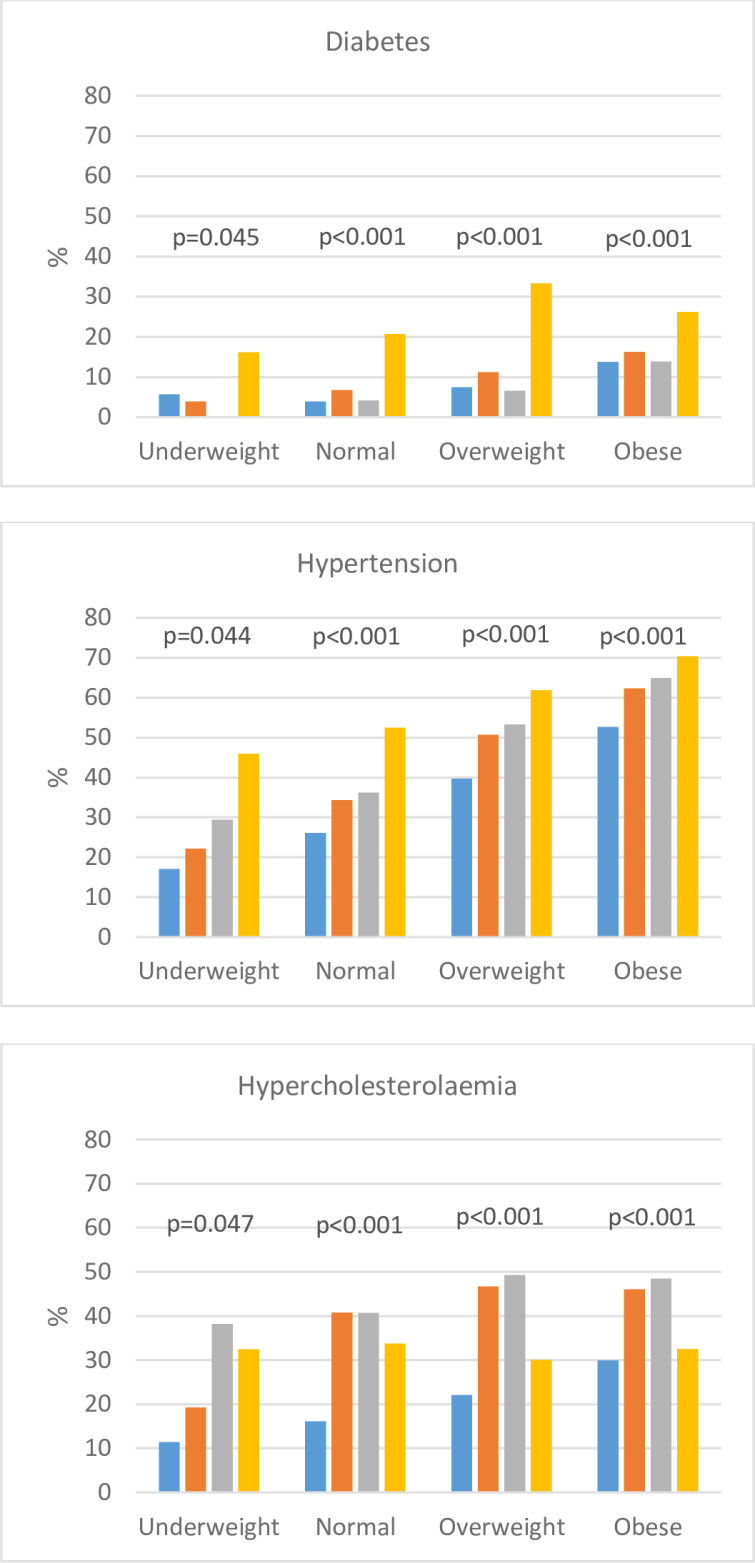
Prevalence of cardio-metabolic diseases by BMI category and population group. Black is designated by a blue square. Coloured is designated by an orange square. White is designated by a grey square. Indian is designated by a yellow square.

Within all BMI categories there were significant differences in the prevalence of diabetes, hypertension and hypercholesterolaemia by population groups (p<0.05) **(Fig 1)**. Across BMI categories, the prevalence of diabetes and hypertension generally increased by population groups. Both were the highest in Indians; diabetes peaked in the overweight (33.3%) while hypertension peaked in the obese (70.4%).

The prevalence of hypercholesterolaemia was higher in participants with BMI ≥25 kg/m^2^, compared with BMI <25 kg/m^2^, in all population groups except in Indians **(Fig 1)**. Hypercholesterolaemia prevalence was highest in whites across all abnormal BMI categories peaking in the overweight (49.3%) and obese (48.5%).

### Associations of cardio-metabolic diseases with gender, population group and BMI category

The adjusted regression models that illustrate the associations of population groups and BMI with cardio-metabolic diseases by gender are presented in **Table 3**. Compared with white men and women, their black African, coloured and Indian counterparts were significantly more likely to have diabetes, with the odds the highest for the Indian men (OR: 4.38, 95% CI: 2.65–7.26) and women (3.64, 2.50–5.32). The odds for hypertension was only significantly higher in coloured compared with white men (1.37, 1.02–1.83), while it was significantly higher in women of all population groups compared with white women; the odds were highest in Indian women (2.38, 1.80–3.13). The odds for hypercholesterolaemia was significantly lower in black African men and women compared with their white counterparts, while it was significantly higher in Indian (1.47, 1.05–2.08) compared with white men.

**Table 3:** Adjusted logistic regression models for association of diabetes, hypertension and hypercholesterolaemia with population groups and body mass index by gender.

	Diabetes		Hypertension		Hypercholesterolaemia	
	Men	Women	Men	Women	Men	Women
**Population group**						
Black African	2.66* (1.70–4.16)	2.10* (1.49–2.96)	1.28 (0.97–1.68)	1.82* (1.46–2.26)	0.64* (0.49–0.84)	0.52* (0.43–0.62)
Coloured	2.28* (1.44–3.60)	2.15* (1.52–3.05)	1.37* (1.02–1.83)	1.94* (1.55–2.42)	0.86 (0.66–1.13)	0.89 (0.74–1.07)
White	1	1	1	1	1	1
Indian	4.38* (2.65–7.26)	3.64* (2.50–5.32)	1.30 (0.91–1.84)	2.38* (1.80–3.13)	1.47* (1.05–2.08)	0.83 (0.65–1.06)
**BMI**						
Underweight	0.75 (0.26–2.17)	0.53 (0.18–1.57)	0.98 (0.53–1.83)	0.59 (0.32–1.08)	0.67 (0.34–1.34)	0.78 (0.46–1.31)
Normal	1	1	1	1	1	1
Overweight	1.41* (1.01–1.96)	1.41* (1.08–1.96)	1.83* (1.48–2.27)	1.51* (1.26–1.82)	1.14 (0.92–1.42)	1.17 (0.99–1.38)
Obese	2.36* (1.66–3.36)	1.76* (1.36–2.28)	3.11* (2.44–3.97)	2.44* (2.04–2.92)	1.53* (1.21–1.94)	1.19* (1.02–1.39)

* p < 0.05

All models were adjusted for variables found to be significant in the univariable analysis. Variables adjusted for include province, physical activity, smoking, alcohol use, fruit/vegetable, high fat and salt intake, and personal and family medical history

Compared with normal weight, overweight and obesity in men and women were significantly associated with diabetes and hypertension. However, obesity, but not overweight, was significantly associated with hypercholesterolaemia in both men and women.

The adjusted regression models that illustrate the associations of gender and BMI with cardio-metabolic diseases by population groups are presented in **Table 4**. Women compared with men were less likely to have diabetes but this was significant only for black Africans (0.64, 0.46–0.89). Black African, coloured and white, but not Indian, women were significantly less likely to have hypertension compared with their male counterparts. In contrast, the odds for hypercholesterolaemia were higher in coloured (1.44, 1.16–1.80) and white (1.47, 1.18–1.84) women, compared with their counterparts. There were no significant gender differences with hypercholesterolaemia for black Africans and Indians.

**Table 4:** Adjusted logistic regression models for association of diabetes, hypertension and hypercholesterolaemia with gender and BMI by population groups.

	Diabetes				Hypertension				Hypercholesterolaemia			
	Black	Coloured	White	Indian	Black	Coloured	White	Indian	Black	Coloured	White	Indian
**Gender**												
Men	1	1	1	1	1	1	1	1	1	1	1	1
Women	0.64** (0.46–0.89)	0.75 (0.53–1.06)	0.68 (0.45–1.01)	0.84 (0.62–1.14)	0.66*** (0.54–0.82)	0.65*** (0.50–0.84)	0.69** (0.53–0.88)	0.99 (0.75–1.32)	0.93 (0.76–1.15)	1.44*** (1.16–1.80)	1.47*** (1.18–1.84)	0.95 (0.74–1.23)
**BMI**												
Underweight	2.54 (0.52–12.40)	0.68 (0.09–5.33)	1	0.81 (0.30–2.21)	0.76 (0.28–2.05)	0.53 (0.19–1.51)	0.44 (0.19–1.05)	1.14 (0.52–2.54)	0.87 (0.29–2.56)	0.33* (0.12–0.90)	0.85 (0.41–1.76)	0.99 (0.48–2.04)
Normal	1	1	1	1	1	1	1	1	1	1	1	1
Overweight	1.55 (0.93–2.59)	1.27 (0.78–2.07)	1.34 (0.77–2.32)	1.79*** (1.28–2.52)	1.41** (1.09–1.83)	1.52** (1.11–2.07)	1.86*** (1.41–2.45)	1.35 (0.98–1.84)	1.29 (0.97–1.70)	1.11 (0.85–1.45)	1.51** (1.18–1.94)	0.83 (0.63–1.11)
Obese	2.47*** (1.54–3.97)	2.04** (1.28–3.23)	2.90*** (1.72–4.88)	1.19 (0.81–1.75)	2.06*** (1.61–2.64)	2.75*** (2.04–3.73)	3.09*** (2.28–4.20)	2.77*** (1.93–3.96)	1.63*** (1.25–2.12)	1.03 (0.80–1.33)	1.55** (1.19–2.03)	0.96 (0.70–1.32)

* p < 0.05

** p < 0.01

*** p ≤ 0.001

All models were adjusted for variables found to be significant in the univariable analysis. Variables adjusted for include province, physical activity, smoking, alcohol use, fruit/vegetable, high fat and salt intake, and personal and family medical history

Compared with normal weight, overweight was significantly associated with diabetes in Indians only (1.79, 1.28–2.52), while obesity was associated with diabetes in black Africans (2.47, 1.54–3.97), coloureds (2.04, 1.28–3.23) and whites (2.9, 1.72–4.88). Overweight and obesity, compared with normal weight, were significantly associated with hypertension in black, coloured and white populations. In Indians, however, obesity (2.77, 1.93–3.96) but not overweight (1.35, 0.98–1.84) was significant.

A generally linear pattern was observed for the association between hypercholesterolaemia and BMI category in black Africans and whites while there was no discernible pattern in Indians or coloureds. Compared with normal weight participants, the odds for hypercholesterolaemia were significant for overweight only in whites (1.51, 1.18–1.94), and for obesity in whites (1.55, 1.19–2.03) and black Africans (1.63, 1.25–2.12).

## Discussion

This study is the first in 3–4 decades, to our knowledge, to examine and compare the risk for the cardio-metabolic diseases of diabetes, hypertension and hypercholesterolaemia across and within the historically defined four South African population groups. Additionally, this study enables a comparison with not only white populations, as is common in the literature, but also across black African, coloured and Indian populations. Furthermore, it provides insights on the differential risks for these cardio-metabolic diseases across and within gender categories, and explores the influence of adiposity on the risk for cardio-metabolic diseases by population group and gender.

### Diabetes

Black Africans, coloureds and Indians had a higher risk for diabetes compared with whites, which accords with the global literature. People of African and South Asian ancestry consistently display a greater risk for diabetes when compared with Europeans [1]. Many studies have shown greater insulin resistance in black Africans [15] and Indians [3, 16], and higher insulin release to maintain normoglycaemia in black Africans compared with whites [15], which likely contributes to their greater susceptibility to diabetes.

Furthermore, Indians have an unfavourable fat distribution compared with other populations [3, 4]. They have a propensity for greater visceral fat accumulation, which is more closely linked to insulin resistance and diabetes [17], and a higher body fat percentage for the same BMI level, age and sex compared with whites and some other population groups [4, 18]. Consequently, people of Asian origin tend to develop diabetes at lower BMI levels compared with other population groups [17–19]. This is likely the reason for the greater diabetes risk in Indians demonstrated at a lower BMI threshold in this study; overweight was significantly associated with diabetes in Indians but not in the other population groups.

The odds for diabetes was highest in Indians compared with the other population groups (4-fold higher vs. 2–2.5-fold higher). Interestingly, this was comparable with Asian Indians in the United States who had a 4-5-fold age-and-BMI adjusted odds for diabetes compared with non-Hispanic whites [20].

Black African women had significantly lower odds for diabetes compared with their male counterparts. This may be due to the differential body fat distribution in men and women, with men having more abdominal and visceral fat accumulation, which is closely linked to insulin resistance. Women have greater peripheral fat deposition, which, in contrast to visceral fat, is associated with improved insulin sensitivity [21], and was protective for diabetes, as reported locally [22].

### Hypertension

The odds for hypertension were significantly higher in coloured compared with white men, while the prevalence of obesity, a key hypertension risk factor, was the highest in coloured compared with other men. Therefore, obesity may play an important role in the development of hypertension in coloured men.

The odds for hypertension in black African and Indian men were not significantly higher than that for white men. This was surprising because hypertension is known to be more prevalent in black Africans and South Asians compared with whites [1]. However, the much lower obesity levels in Indian (17.6%) compared with white men (28.6%) in this study may partly account for the absence of a greater risk for hypertension in the former.

In women, the odds for hypertension were higher in black Africans, coloureds and Indians compared with whites. The much higher obesity levels in black African (58%) and coloured women (47.2%) compared with white women (27.1%) may account for their greater hypertension odds. Indian women, however, had similar obesity levels (29.9%) compared with white women but had the highest odds for hypertension by population group. Furthermore, hypertension prevalence was high in underweight (46%) and normal weight (52.5%) Indians, which highlights the multi-factorial aetiology of hypertension in this population.

Black African, coloured and white, but not Indian, women were significantly less likely to have hypertension compared with their male counterparts. Globally, the trends for hypertension by gender are inconsistent with some studies showing a higher prevalence in either gender or others reporting no difference [23]. Systematic reviews, including those that focused on Africa, reported comparable hypertension prevalence in men and women [24–27]; some with an insignificant male preponderance [25–27]. Studies in South Africa reported a similar to slightly higher hypertension prevalence in women compared with men [28–31].

### Hypercholesterolaemia

Black African men and women were significantly less likely to have hypercholesterolaemia compared with their white counterparts, which accords with reports from other South African studies [32, 33] and internationally [1]. Although black Africans exhibited a lower risk for hypercholesterolaemia compared with other population groups and have demonstrated athero-protective lipid profiles in the past [33, 34], more recent evidence points to worsening lipid profiles in urban black Africans [35]. The significant association of obesity with hypercholesterolaemia and the rising hypercholesterolaemia prevalence by BMI category in black Africans supports the influence of unhealthy lifestyles and ensuing obesity in the development of the condition in this population.

Obesity was significantly related to hypercholesterolaemia in men and women, consistent with the positive association of BMI with raised total cholesterol in the Heart of Soweto study [33]. Nevertheless, by population group, the association of obesity with hypercholesterolaemia was significant only for black Africans and whites. The absence of an association between adiposity and hypercholesterolaemia in coloureds and Indians in this study suggests a multi-factorial aetiology in the development of lipid abnormalities and may require further investigation. Interestingly, Seedat and colleagues also noted no association between obesity and hypercholesterolaemia in Indian South Africans [36].

Indian men had the highest odds for hypercholesterolaemia with a 47% greater risk than that for white men. This accords with the Heart of Soweto study where the odds for hypercholesterolaemia was highest for Indian ethnicity [33]. Furthermore, Norman and colleagues reported the highest cholesterol attributable mortality rates in Indians, followed by whites and then coloureds in South Africa [32].

The strengths of this study include the large number of participants and that few individuals across all population groups in this cohort were likely to be migrants allowing for a robust comparison of the data. Moreover, this is one of few studies conducted nationally to compare cardio-metabolic diseases across all four population groups. Despite this being a convenience sample and the absence of rural residents, both limitations of this study, the large number of participants provides a unique opportunity allowing for comparisons across and within population groups and gender categories. Another limitation is the cross-sectional study design which precludes any causal associations with the cardio-metabolic diseases. That RBG levels were used to diagnose diabetes without determining the presence of hyperglycaemia related symptoms could have overestimated the prevalence of diabetes in this sample.

## Conclusions

The cardio-metabolic diseases of diabetes, hypertension and hypercholesterolaemia were differentially associated with population groups and gender in South Africa, as well as with adiposity within some of these subgroups. In particular, compared with their counterparts, Indian men had the greatest risk for diabetes and hypercholesterolaemia, while Indian women had the highest odds for diabetes and hypertension. Black African men and women had the lowest likelihood for hypercholesterolaemia compared with their counterparts. Adiposity was generally associated with these cardio-metabolic diseases by gender and population groups, except for hypercholesterolaemia in coloureds and Indians.

Future research could identify the modifiable behavioural risk factors that differentially influence the development of cardio-metabolic diseases in these populations. This will enable the development of culturally tailored prevention strategies and may contribute to better management of CVDs in the disadvantaged South African populations.

## Supporting information

S1 Table Socio-demographic characteristics (N, %), lifestyle behaviours and medical history presented by body mass index categories. (DOCX)

S2 Table Cardio-metabolic risk factors presented by body mass index categories. (DOCX)
