# Supplementary material for: Differential associations of cardio-metabolic diseases by population group, gender and adiposity in South Africa
Source: PLoS One. 2018 Sep 27;13(9):e0202899. doi: 10.1371/journal.pone.0202899 (PMC6160009; doi:10.1371/journal.pone.0202899)
Supplement: S1 Table — (DOCX) [file pone.0202899.s001.docx]

**S1 Table: Socio-demographic characteristics (N, %), lifestyle behaviours and medical history presented by body mass index categories**

|  | **Men** | | | | | **Women** | | | | |
| --- | --- | --- | --- | --- | --- | --- | --- | --- | --- | --- |
|  | **Underweight** | **Normal weight** | **Overweight** | **obese** | **p-value** | **Underweight** | **Normal weight** | **Overweight** | **obese** | **p-value** |
| Number, % | **59 (2.4)** | **798 (32.1)** | **987 (39.8)** | **638 (25.7)** |  | **81 (1.6)** | **1322 (25.4)** | **1530 (29.3)** | **2280 (43.7)** |  |
| **Socio-demographic** |  |  |  |  |  |  |  |  |  |  |
| Age, mean, SD | 40.7 (18.8) | 45.0 (17.8) | 49.1 (15.8) | 49.1 (14.1) | <0.001 | 46.8 (22.1) | 46.2 (18.9) | 49.6 (16.7) | 49.3 (14.1) | <0.001 |
| **Province** |  |  |  |  | <0.001 |  |  |  |  | <0.001 |
| Western Cape | 17 (1.9) | 278 (30.5) | 357 (39.2) | 259 (28.4) |  | 32 (1.5) | 477 (21.5) | 618 (27.8) | 1093 (49.2) |  |
| Eastern Cape | 6 (1.6) | 87 (24.0) | 164 (45.2) | 106 (29.2) |  | 5 (0.8) | 198 (30.3) | 192 (29.4) | 258 (39.5) |  |
| Kwazulu-Natal | 25 (4.2) | 244 (41.0) | 207 (34.7) | 120 (20.1) |  | 34 (2.6) | 390 (29.9) | 424 (32.5) | 457 (35.0) |  |
| Free State | 2 (1.7) | 37 (31.9) | 42 (36.2) | 35 (30.2) |  | 0 (0.0) | 47 (29.0) | 43 (26.5) | 72 (44.5) |  |
| Gauteng | 9 (1.8) | 152 (30.6) | 217 (43.8) | 118 (23.8) |  | 10 (1.1) | 210 (24.1) | 253 (29.0) | 399 (45.8) |  |
|  |  |  |  |  |  |  |  |  |  |  |
| **Lifestyle behaviours** |  |  |  |  |  |  |  |  |  |  |
| Physical activity >150 min/week | 27 (45.8) | 437 (54.8) | 501 (50.8) | 277 (43.4) | <0.001 | 37 (45.7) | 660 (49.9) | 712 (46.5) | 970 (42.5) | <0.001 |
| Smoking | 24 (40.7) | 253 (31.7) | 216 (21.9) | 125 (19.6) | <0.001 | 21 (25.9) | 212 (16.0) | 219 (14.3) | 229 (10.0) | <0.001 |
| Alcohol use | 23 (39.0) | 325 (40.7) | 403 (40.8) | 270 (42.3) | 0.902 | 25 (30.9) | 371 (28.1) | 338 (22.1) | 389 (17.1) | <0.001 |
| Problem drinkers^a^ | 23 (100.0) | 325 (100.0) | 398 (98.8) | 270 (100.0) | 0.851 | 25 (100.0) | 369 (99.5) | 335 (99.1) | 385 (99.0) | <0.001 |
| **Food intake** |  |  |  |  |  |  |  |  |  |  |
| ≥5 fruit & vegetables/day | 24 (40.7) | 424 (53.1) | 495 (50.2) | 317 (49.7) | 0.200 | 35 (43.2) | 721 (54.5) | 896 (58.6) | 1368 (60.0) | 0.001 |
| High fat foods | 36 (61.0) | 451 (56.5) | 524 (53.1) | 374 (58.6) | 0.121 | 35 (43.2) | 565 (42.7) | 689 (45.0) | 1230 (54.0) | <0.001 |
| High salt foods | 35 (59.3) | 425 (53.3) | 511 (51.8) | 363 (56.9) | 0.179 | 38 (46.9) | 555 (42.0) | 664 (43.4) | 1255 (55.0) | <0.001 |
|  |  |  |  |  |  |  |  |  |  |  |
| **Personal medical history** |  |  |  |  |  |  |  |  |  |  |
| Cardiac/stroke | 8 (13.6) | 96 (12.0) | 140 (14.2) | 83 (13.0) | 0.607 | 14 (17.3) | 172 (13.0) | 194 (12.7) | 274 (12.0) | 0.552 |
| Diabetes | 5 (8.5) | 75 (9.4) | 116 (11.8) | 101 (15.8) | 0.002 | 4 (5.0) | 101 (7.6) | 200 (13.1) | 316 (13.9) | <0.001 |
| Hypertension | 11 (18.6) | 141 (17.7) | 282 (28.6) | 235 (36.8) | <0.001 | 15 (18.5) | 282 (21.3) | 482 (31.5) | 827 (36.3) | <0.001 |
| **Family medical history**^b^ |  |  |  |  |  |  |  |  |  |  |
| Father | 7 (11.9) | 59 (7.4) | 96 (9.7) | 61 (9.6) | 0.262 | 10 (12.4) | 146 (11.0) | 148 (9.7) | 226 (9.9) | 0.551 |
| Mother | 5 (8.5) | 57 (7.1) | 63 (6.4) | 55 (8.6) | 0.387 | 7 (8.6) | 118 (8.9) | 167 (10.9) | 232 (10.2) | 0.343 |
|  |  |  |  |  |  |  |  |  |  |  |

^a^Among alcohol consumers, men who drank ≥2 units/day or women who drank ≥1 unit/day;

^b^Any cardiac problem or stroke in a father before 55 years of age or in a mother before 65 years of age
