# Supplementary material for: Differential associations of cardio-metabolic diseases by population group, gender and adiposity in South Africa
Source: PLoS One. 2018 Sep 27;13(9):e0202899. doi: 10.1371/journal.pone.0202899 (PMC6160009; doi:10.1371/journal.pone.0202899)
Supplement: S2 Table — (DOCX) [file pone.0202899.s002.docx]

**S2 Table: Cardio-metabolic risk factors presented by body mass index categories ***

|  | **Men** | | | | | **Women** | | | | |
| --- | --- | --- | --- | --- | --- | --- | --- | --- | --- | --- |
|  | **Underweight** | **Normal weight** | **Overweight** | **obese** | **p-value** | **Underweight** | **Normal weight** | **Overweight** | **obese** | **p-value** |
| Number | **59 (2.4)** | **798 (32.1)** | **987 (39.8)** | **638 (25.7)** |  | **81 (1.6)** | **1322 (25.4)** | **1530 (29.3)** | **2280 (43.7)** |  |
| BMI, mean, SD (kg/m^2^) | 17.7 (1.2) | 22.6 (2.0) | 27.4 (1.7) | 34.1 (4.1) |  | 18.2 (4.1) | 22.6 (2.0) | 27.5 (1.8) | 36.1 (5.1) |  |
| **Blood pressure (BP)** |  |  |  |  |  |  |  |  |  |  |
| Mean, SD (mmHg): |  |  |  |  |  |  |  |  |  |  |
| Systolic BP | 125.7 (14.2) | 131.9 (17.1) | 136.8 (17.6) | 140.0 (17.9) | <0.001 | 122.9 (21.1) | 127.9 (20.3) | 133.0 (19.4) | 135.6 (20.1) | <0.001 |
| Diastolic BP | 75.5 (11.2) | 76.4 (11.5) | 79.6 (11.8) | 82.3 (11.5) | <0.001 | 72.4 (11.8) | 74.3 (11.0) | 77.5 (11.0) | 80.4 (11.7) | <0.001 |
| SBP ≥140 mmHg or DBP ≥90 mmHg or on treatment, % | 12 (20.3) | 231 (29.0) | 420 (42.6) | 332 (52.0) | <0.001 | 18 (22.2) | 336 (25.4) | 516 (33.8) | 944 (41.4) | <0.001 |
| Known hypertension on treatment,%** | 11 (18.6) | 141 (17.7) | 282 (28.6) | 235 (36.8) | <0.001 | 15 (18.5) | 282 (21.3) | 482 (31.5) | 827 (36.3) | <0.001 |
| % of known hypertension with BP <140/90 mmHg | 8 (72.7) | 71 (50.4) | 118 (41.8) | 93 (39.6) | <0.001 | 5 (33.3) | 135 (47.9) | 224 (46.5) | 367 (44.4) | <0.001 |
| **Random blood glucose** |  |  |  |  |  |  |  |  |  |  |
| Mean, SD (mmol/l) | 6.1 (3.4) | 6.2 (2.4) | 6.5 (3.0) | 6.9 (2.8) | <0.001 | 6.0 (3.0) | 6.1 (2.5) | 6.4 (2.6) | 6.5 (2.6) | <0.001 |
| RBG: 7.0-11.0 mmol/l | 8 (13.6) | 124 (15.5) | 176 (17.8) | 160 (25.1) | <0.001 | 9 (11.1) | 168 (12.7) | 259 (16.9) | 402 (17.6) | <0.001 |
| RBG ≥11.1 or known diabetes, % | 5 (8.5) | 78 (9.8) | 136 (13.8) | 123 (19.3) | <0.001 | 4 (4.9) | 111 (8.4) | 206 (13.5) | 347 (15.2) | <0.001 |
| Newly diagnosed diabetes, % | 0 (0.0) | 3 (3.8) | 20 (14.7) | 22 (17.9) | <0.001 | 0 (0.0) | 10 (9.0) | 6 (2.9) | 31 (8.9) | 0.013 |
| Known diabetes, %** | 5 (100.0) | 75 (96.2) | 116 (85.3) | 101 (82.1) | 0.002 | 4 (100.0) | 101 (91.0) | 200 (97.1) | 316 (91.1) | <0.001 |
| % of known diabetes with RBG <7.0 mmol/l | 1 (20.0) | 23 (30.7) | 43 (37.1) | 30 (29.7) | 0.654 | 2 (50.0) | 45 (44.6) | 67 (33.5) | 112 (35.4) | 0.219 |
| **Total cholesterol** |  |  |  |  |  |  |  |  |  |  |
| Mean, SD (mmol/l) | 4.3 (0.9) | 4.5 (1.1) | 4.7 (1.2) | 4.9 (1.4) | <0.001 | 4.8 (1.4) | 4.8 (1.3) | 4.8 (1.3) | 4.8 (1.3) | 0.439 |
| Total cholesterol >5 mmol/l, % | 11 (18.6) | 213 (26.7) | 315 (31.9) | 243 (38.1) | <0.001 | 24 (29.6) | 458 (34.6) | 596 (39.0) | 856 (37.5) | 0.051 |
| **Prevalence of any of the 3 cardio-metabolic abnormalities** |  |  |  |  | <0.001 |  |  |  |  | <0.001 |
| 1 abnormality | 23 (39.0) | 297 (37.2) | 423 (42.9) | 290 (45.5) |  | 29 (35.8) | 491 (37.1) | 592 (38.7) | 923 (40.5) |  |
| 2 abnormalities | 5 (8.5) | 124 (15.5) | 247 (25.0) | 198 (31.0) |  | 8 (9.9) | 225 (17.0) | 367 (24.0) | 637 (27.9) |  |
| 3 abnormalities | 1 (1.7) | 16 (2.0) | 24 (2.4) | 35 (5.5) |  | 2 (2.5) | 33 (2.5) | 72 (4.7) | 106 (4.7) |  |
|  |  |  |  |  |  |  |  |  |  |  |

*Data presented as N (%) or mean (SD);

**Among those with hypertension or diabetes, the proportion that was known
